# The function of endocytosis in Wnt signaling

**DOI:** 10.1007/s00018-017-2654-2

**Published:** 2017-09-14

**Authors:** Lucy Brunt, Steffen Scholpp

**Affiliations:** 1 Living Systems Institute, School of Biosciences, College of Life and Environmental Science, University of Exeter, Exeter, EX4 4QD UK; 2 Institute of Toxicology and Genetics (ITG), Karlsruhe Institute of Technology (KIT), Karlsruhe, Germany

**Keywords:** Wnt, Endocytosis, Signal activation, Planar cell polarity, Cytonemes, Exosomes

## Abstract

Wnt growth factors regulate one of the most important signaling networks during development, tissue homeostasis and disease. Despite the biological importance of Wnt signaling, the mechanism of endocytosis during this process is ill described. Wnt molecules can act as paracrine signals, which are secreted from the producing cells and transported through neighboring tissue to activate signaling in target cells. Endocytosis of the ligand is important at several stages of action: One central function of endocytic trafficking in the Wnt pathway occurs in the source cell. Furthermore, the β-catenin-dependent Wnt ligands require endocytosis for signal activation and to regulate gene transcription in the responding cells. Alternatively, Wnt/β-catenin-independent signaling regulates endocytosis of cell adherence plaques to control cell migration. In this comparative review, we elucidate these three fundamental interconnected functions, which together regulate cellular fate and cellular behavior. Based on established hypotheses and recent findings, we develop a revised picture for the complex function of endocytosis in the Wnt signaling network.

## Introduction

The Wnt signaling network comprises a multitude of ligands, receptors, co-receptors, and intracellular effectors to regulate fundamental cellular decisions in development and tissue homeostasis. The current consensus is that there are 19 ligands of the Wnt family, 10 Frizzled (Fzd) receptors, and a handful co-receptors including lipoprotein receptor-related proteins (Lrps), receptor tyrosine kinases, etc. The cumulative evidence obtained over the last decade has demonstrated that this family of glycoproteins acts as intercellular paracrine, juxtacrine, and endocrine signals in all multicellular organisms [1]. Signaling by Wnt is an important process that guides cell polarity, cell proliferation, cell migration, and cell fate specifications during embryogenesis and tissue maturation, regeneration, and renewal [2].

One crucial and highly investigated branch of the Wnt network is canonical Wnt signaling which regulates the concentration of the transcriptional co-activator β-Catenin. β-Catenin and the transcription factors of the Tcf/Lef family control gene expression programs. In the Wnt-off state, β-Catenin protein concentration is kept low in the cytoplasm by the so-called destruction complex. This multimeric complex is composed of the scaffolding protein Axin1, Casein kinase 1 (Ck1), and Glycogen synthase kinase 3 (Gsk3) and other proteins [3]. Ck1 and Gsk3 sequentially phosphorylate the N-terminal region of β-Catenin, which results in β-Catenin degradation. This continuous degradation prevents accumulation and translocation of β-Catenin to the nucleus. In the Wnt-on state, Wnt ligand binds to seven-pass transmembrane Fzd receptors and its co-receptor, low-density lipoprotein receptor-related protein 5/6 (Lrp5/6). The formation of a Wnt–Fzd–Lrp complex leads to the recruitment of the destruction complex to the receptors. Subsequently, Lrp6 is phosphorylated leading to inactivation of Gsk3 and blockage of β-Catenin phosphorylation, and thereby stabilization of β-Catenin. The stabilized β-Catenin can then accumulate in the nucleus where it interacts with Tcf/Lef and activates Wnt-dependent target gene expression. Alternate Wnt receptors such as G-protein-coupled receptor 124 (GPR124) have been suggested as co-activators of the Wnt/β-Catenin pathway in vitro and in mouse following Wnt7 stimulation [4].

The main β-catenin-independent branches of the Wnt network comprise the planar cell polarity (PCP) pathway and the Wnt–Ca^2+^ pathway [5]. The PCP pathway is involved in regulation of the cytoskeleton, cell polarity, and cell migration in the *Drosophila* wing, eye, abdomen, and notum and during vertebrate gastrulation and neurulation, to name just a few processes [6]. The best-described signaling molecules activating the β-catenin-independent pathway are Wnt5 and Wnt11. Like the β-catenin-dependent pathway, these Wnt ligands bind to Fzd receptors which may explain their suggested antagonistic crosstalk with the β-catenin-dependent pathway. In addition, receptor tyrosine kinase-like orphan receptor 2 (Ror2), protein tyrosine kinase 7 (Ptk7), receptor tyrosine kinase (Ryk), and muscle skeletal receptor tyrosine kinase (Musk) have been suggested as β-catenin-independent co-receptors. Frizzled receptors, together with several effectors such as Van Gogh/Prickle and Dishevelled (Dvl), are often asymmetrically localized in cells regulating polarization. When PCP signaling is active, it can locally activate both Rho and Rac signaling to control actomyosin-mediated cytoskeletal changes and thus, cell extensions and cell migration in invertebrates and vertebrates. The Wnt–Ca^2+^ pathway is involved in inflammation and neurodegeneration by activating phospholipase C and inositol-1,4,5-trisphosphate, triggering intracellular Ca^2+^ release [7]. The Wnt–Ca^2+^ pathway can also be activated by Wnt ligands such as Wnt9a by binding Polycystin1, an atypical G-protein-coupled receptor, which mediates TRPP2 calcium ion channel influx, important for pronephric tubule formation in *Xenopus* [8].

Receptor-mediated endocytosis is a specific process which enables cells to take up molecular complexes such as ligand–receptor complexes. The uptake of transferrin by the transferrin receptor has come to define Clathrin-dependent internalization [9]. In addition, there is also the possibility of uptake of ligand–receptor complexes in a Clathrin-independent manner, which most often involves Caveolins [10]. These internalization routes are intimately linked to Wnt signaling [11, 12]. In a simplified view, Clathrin-dependent endocytosis promotes PCP signaling, whereas Clathrin-independent endocytosis promotes β-catenin-dependent signaling [13]. Indeed, there is supporting evidence that PCP components together with Syndecans are taken up by Clathrin-mediated endocytosis [14], whereas Wnt3a–Lrp6 is internalized through a Caveolin-mediated route [15, 16]. However, there is also growing evidence that Clathrin-mediated endocytosis of Wnt and Frizzled receptors enhances β-catenin-dependent signaling [17–20]. In summary, it is still unclear how endocytosis and Wnt signaling is intertwined.

In this review, we will discuss the current picture regarding endocytosis and Wnt signaling. In detail, we will elucidate critical functions of endocytosis during signal activation of the Wnt pathway. We first elucidate how endocytosis of Wnt/Wg together with its chaperones is involved in the secretion of the ligand. We will then discuss how endocytosis can act as a prerequisite to activate signaling in the target cells. Finally, the role of Wnt endocytosis in cell migration will be addressed.

## Endocytosis in the Wnt source cell

**Fig. 1:**
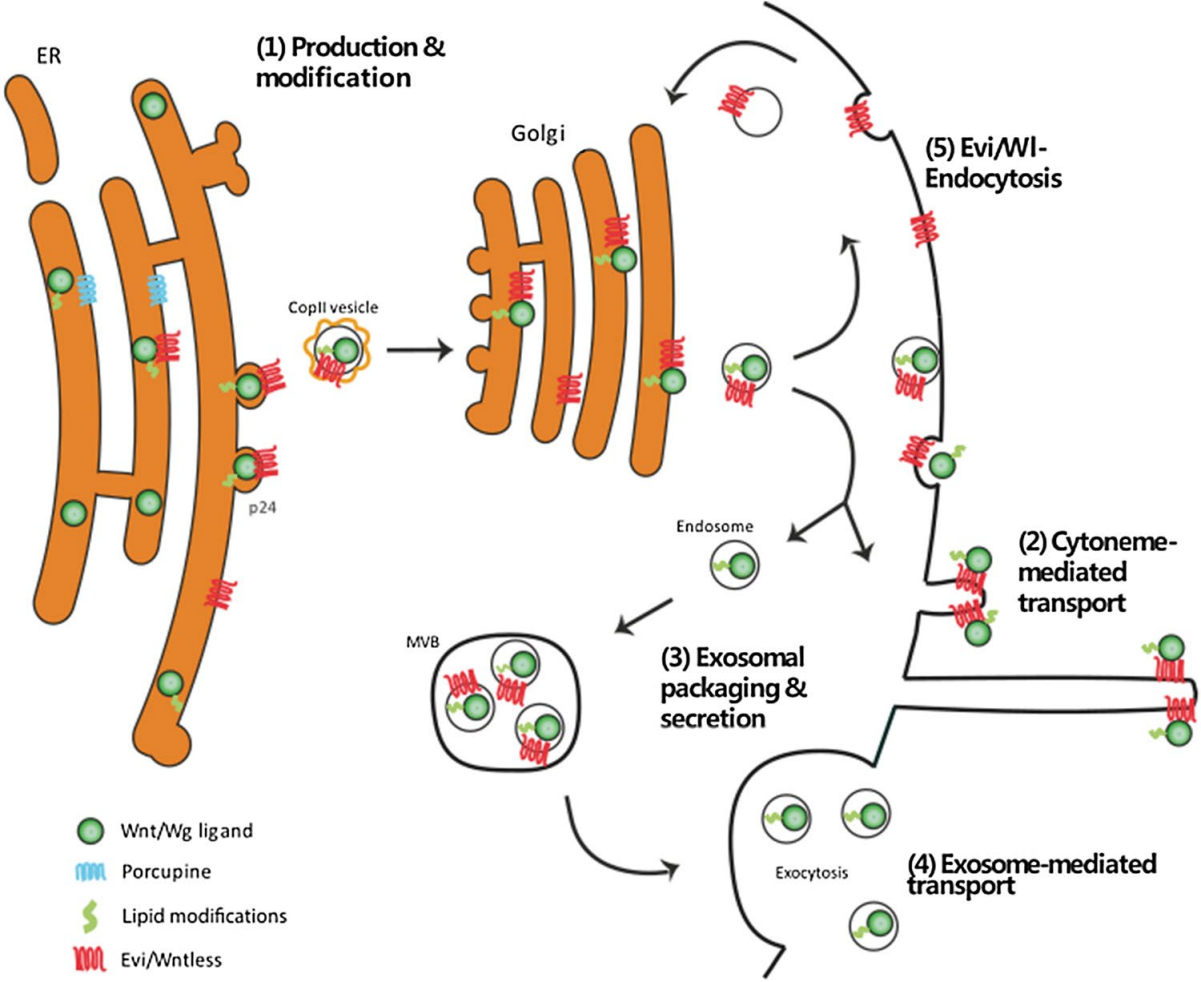
Endocytosis-regulated Wnt/Wg secretion. After formation and lipid modification of the ligand (1), Evi/Wl transports Wnt/Wg to the plasma membrane. The ligand induces either a cytoneme (2) or it gets re-endocytosed and packaged in exosomes (3) for the subsequent secretion (4). Re-endocytosis and transport of Evi/Wl to the Golgi close the loop (5)

Classically, the point of signaling pathway initiation occurs at the point of secretion of extracellular signals from a cell group, which then propagate through the neighboring tissue to regulate the behavior of the signal-receiving cells. However, prior to the secretion process, Wnt/Wg undergoes modification, sorting, and packaging within the source cell, and endocytosis plays has a pivotal role in Wnt ligand presentation at the membrane (Fig. 1(1)).

In the ligand-producing cells prior to secretion, Wnt/Wg is integrated into the endoplasmic reticulum (ER) lumen where it is posttranslationally lipid modified and glycosylated (Fig. 1(1)). These posttranslational modifications of the Wnt molecules are a prerequisite for secretion and signaling [21–23]. Porcupine (Porcn) is a multipass transmembrane *O*-acyltransferase in the ER that is important for palmitoylation and maturation of Wnt/Wg [24–26]. Following displacement from the ER, the Wnt ligand is transported to the membrane for secretion with the Wnt cargo receptor, Evenness interrupted/Wntless (Evi/Wl). The interaction of Evi/Wl with the ligand is necessary for secretion [27–29].

After membrane presentation, the Wg ligand was found to be re-endocytosed into the producing cells in *Drosophila* [30]. Data suggest that final secretion of Wg ligand is reliant on dynamin-dependent endocytosis. The Wg protein was shown to accumulate in source cells in the *shibire* mutant fly carrying a temperature-sensitive mutation in Dynamin [31]. These data suggest that the subsequent transport of Wnt/Wg to the apical or basolateral membrane is a directed process depending on posttranslational glycosylation of the ligand [32]. Dynamin-dependent endocytosis of Wg from the apical surface of *Drosophila* wing disk epithelium is required before it is transported to the basolateral surface [33]. Therefore, endocytosis is an important process for subsequent Wg ligand secretion.

Methods of Wnt/Wg secretion include transport on signaling filopodia—the so-called cytonemes—in *Drosophila* and chick, frog, and fish (Fig. 1(2)) [34–38]. For example, Wnt8a is placed on the tip of cytonemes to be translocated to the neighboring cells in zebrafish [34]. Specifically, at these tips, Wnt8a co-localizes with Evi/Wl during transport. Besides cytoneme-based transport, there is evidence for a further transport mechanism on exosomes [39] (Fig. 1(3), (4)). Recent data suggest that apically released Wnt3a and Wnt11 can be found on CD63-positive exosomes, whereas basolateral secreted Wnt3a is co-fractioning with TSG101 exosomes [40].

The secretion mechanism of exocytosis requires the passage of secretory cargo from endosomes into multivesicular bodies (MVBs) (Fig. 1(3)). There is evidence that Evi/Wl and Wnt/Wg proteins reach this endosomal compartment together before final secretion. Furthermore, the impaired functions of proteins of the ESCRT and the Snare family, such as HGS and Ykt6, which participate in the genesis of the MVB, inhibit Wnt3 secretion [41]. In the neuromuscular junction of *Drosophila*, Wg and Evi/Wl are released together with exosomes [42]. This process requires the endosomal regulator Rab11 and its effector Myosin5A [43]. Exosome-based Wnt/Wg transport is, therefore, impaired after endocytic inhibition. Notably, cytoneme-based transport is also reduced after endocytic blockage [44] although Wnt cytonemes would not need endocytosis for their generation. There is a possible simple explanation for this observation: the carrier Evi/Wl also undergoes endocytic recycling from the membrane of Wnt/Wg-producing cells (Fig. 1(5)) [45, 46]. Thus, endocytosis of the Wg/Wnt ligand and the Wnt transporter Evi/Wl is an essential and evolutionarily conserved mechanism required for continuous Wnt secretion, regardless of the subsequent mode of transport—by cytonemes or exosomes.

## The endocytic route of Wnt in the receiving cell

**Fig. 2:**
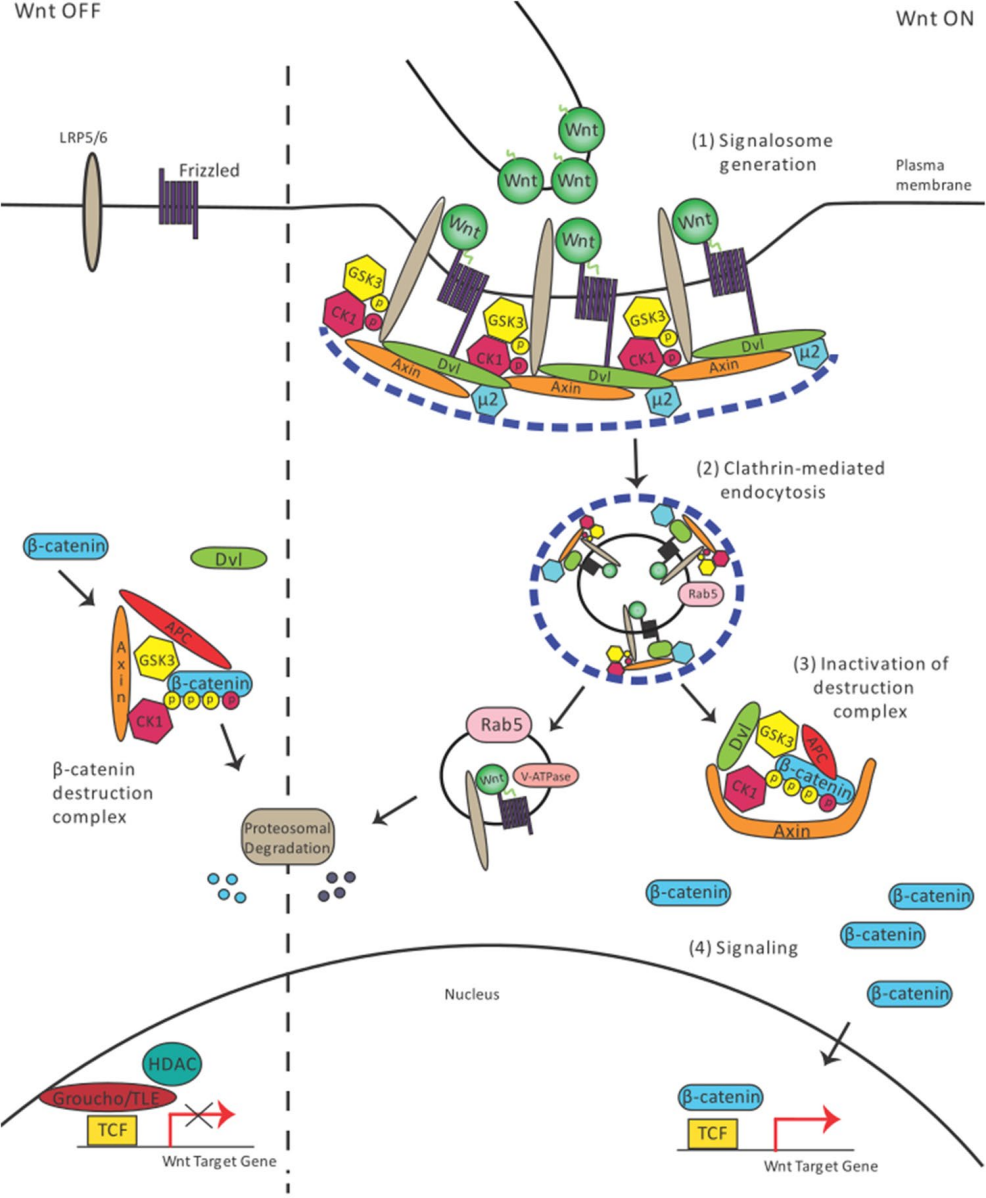
Endocytosis mechanism essential for Wnt/β-catenin signaling. In the Wnt off state, β-catenin is continuously degraded. Wnt presentation to the receiving cell leads to signalosome formation (1). The signalosome is taken up into the cell (2). Disassembly of the Clathrin lattice (blue dashed line) leads to separation of the endocytic vesicles carrying the ligand and the β-catenin/destruction complex, which becomes deactivated (3). Next, β-catenin accumulates and starts to regulate the transcription program in the nucleus (4)

In the Wnt/β-Catenin-dependent signaling branch, endocytosis plays a further noteworthy role. Following secretion and transport, Wnt/Wg binds to specific transmembrane receptors at the cell surface of the responding cells, which leads to clustering of a multimeric complex of several receptors and ligands, termed the signalosome (Fig. 2(1)) [15, 47]. The formation of the signalosome triggers signal initiation by binding of Lrp6, Dvl, and Gsk3, which inhibits Gsk3-mediated phosphorylation of β-Catenin (Fig. 2(3), (4)). However, the mechanism of how endocytosis affects signaling has not been fully elucidated.

Previous studies have indicated that endocytosis plays an important role in Wnt/β-Catenin signaling in cultured mammalian L-cells [17]. Studies using super-resolution fluorescence microscopy suggested that Clathrin is a prerequisite for the formation of the Lrp6-signalosome, which is several hundreds of nanometers in size [48]. Interestingly, recent in vivo studies demonstrated that the µ2 subunit of the Clathrin-specific adaptor protein AP2 is required for Lrp6-signalosome function in zebrafish (Fig. 2(1), (2)) [19].

Studies have used pharmacological reagents such as monodansylcadaverine and chlorpromazine to inhibit Clathrin-mediated internalization [17]. The assembly of Clathrin lattices can also be blocked by hypertonic sucrose, and dominant negative Dynamin can constrain endogenous dynamin-dependent internalization [18]. These treatments and genetic manipulations all lead to reduced Wnt/β-Catenin signaling, as shown by a reduction of β-Catenin stability. In addition, the knockdown of Rab5 or Dynamin using RNA interference approaches in cell culture leads to significantly reduced TOPFlash Wnt reporter activation. A multipurpose drug, Suramin, also blocked Wnt endocytosis, which significantly reduced TOPFlash Wnt reporter activity and β-Catenin stability in HEK293 and L-cells [49]. The functional blockage of the actin modulators, FilaminA and Formin2, impaired Lrp6 endocytosis and reduced β-Catenin accumulation in the nucleus of neural progenitor cells [50]. The importance of endocytosis was also demonstrated by experiments with the human HEK293T cell line. Blocking internalization of the signalosome in these cells led to reduced signaling [16]. These results suggest that internalization of Wnt is required for the activation of the pathway (Fig. 2(2)) [12, 51]. The use of a new generation of small molecule inhibitors blocking Dynamin activity in *Drosophila* S2R+ cells led to a similar conclusion [52].

Endocytosis can also regulate Wnt signal activation by targeting signalosome components for degradation. The interaction of the Clathrin adaptor Dab2 with Lrp6 leads to the degradation of Lrp6 via Clathrin-mediated endocytosis and leads to reduction of signaling [53]. Sorting Nexin27 (SNX27), which regulates Clathrin-mediated endocytosis, was found to interact with the Wnt receptor, Fzd7, promoting Fzd7 internalization and degradation, which led to reduced TOPFlash Wnt reporter activation [54]. A genomewide siRNA screen revealed that deubiquitinase USP6 overexpression could activate Wnt signaling by inhibition of Fzd7 endocytosis and its subsequent targeting for degradation [55]. Therefore, endocytosis plays an important regulatory role in Wnt signaling activation.

Further studies focused on the mode of endocytosis utilized by members of the Wnt signaling family. It has been suggested that Wnt and Lrp6 are regulated via Caveolin-mediated endocytosis. However, the Wnt antagonist Dkk1 that binds to Lrp6 is internalized via Clathrin-mediated endocytosis [56]. Caveolin and Rab8b are required for β-Catenin stabilization and signaling suggesting that Caveolae-mediated endocytosis positively influences Wnt signaling [57]. Recent publications suggest that the phosphorylation status of Lrp6 influences the endocytic route of the ligand–receptor complex. The phosphorylation of tyrosine in the Lrp6 intracellular domain attenuates Wnt signaling by reducing signalosome formation in Caveolin-positive lipid rafts [58]. Furthermore, the phosphorylation of a serine residue at position 1579 within the intracellular domain of Lrp6 by CK2 in response to Wnt ligand promotes binding of Dab2 to Lrp6. This cascade leads to Clathrin-dependent endocytosis and subsequent degradation. Thus, when this serine site is mutated, Lrp6 is recruited to Caveolae, leading to delayed signaling [53].

In summary, there is accumulating evidence that endocytosis of the ligand–receptor complex facilitates Wnt/β-Catenin signaling [12]. However, the mechanistic link between Clathrin- or Caveolin-mediated endocytosis and activation of the signal transduction cascade requires further investigation. Analysis of intact tissues and organisms would be preferable to fully understand the complexity of the regulation of these entry routes.

How is endocytosis linked to Wnt signaling in the receiving cell? At present, there are four alternative ways to explain the requirement for endocytosis in Wnt signal activation.

(i) Signal activation by endosomal acidification
  The importance of endocytosis for Wnt/β-Catenin signal transduction may be explained by the acidification of the Wnt-receptor complex, which is required for its activation (Fig. 2(3)). Upon Wnt stimulation, the signalosome complex is endocytosed and directed to acidic early endosomes. Functional analysis of the core component for acidification, the vesicle membrane V-ATPase, suggested that Wnt signaling depends on acidification [59]. It was found that Prorenin receptor acts as an adaptor protein between Lrp6 and Vacuolar H+-ATPase to promote Wnt-receptor endocytosis, phosphorylation, and Wnt/β-Catenin signaling activation. A recent study proposed that Rabconnectin3a associates with subunits of the V-ATPase and promotes endosomal maturation to coordinate Wnt signaling and intracellular trafficking of Wnt receptors in zebrafish neural crest [60]. These data suggest that the Wnt-receptor Lrp6 must acidified during endocytosis for subsequent activation by phosphorylation to initiate and maintain signaling.
(ii) Signal activation by sequestering of Gsk3
  An additional theory regarding the role of endocytosis during Wnt/β-Catenin signal activation is based on the observation that after Wnt activation, Gsk3 was found in MVBs [61]. The sequestration of Gsk3 from the cytoplasm was suggested to limit β-Catenin phosphorylation and proteolysis [12]. A recent study demonstrated that the interaction between E-cadherin and p120 is required for clustering of Lrp6-signalosomes. These complexes are internalized in MVBs through their interaction with Caveolin [62]. However, the interpretation that sequestering of Gsk3 is a prerequisite for Wnt signaling raises many questions [63]. These experiments were conducted using a truncated form of a constitutively active Lrp6, and further evidence is needed to determine whether the full-length Lrp6 can recruit Gsk3 into MVBs as well. In addition, only 3–5% of the endogenous Gsk3 pool is associated with Axin [64]. However, the observed relocalization suggests that the majority of the co-expressed Gsk3 is routed to MVBs [61]. Finally, a recent study in *Drosophila* failed to provide evidence that MVB biogenesis makes impact on signaling [52]. The same study showed that reduced signaling by blocking endocytosis cannot be rescued by pharmacological inhibition of Gsk3 activity [52]. In summary, there is evidence suggesting that endocytosis is important for Wnt signaling in a Gsk3-independent manner.
(iii) Ubiquitin ligases regulate signal
  Wnt signaling activity may also be controlled through regulation of Wnt-receptor ubiquitination, endocytosis, and subsequent targeting for degradation. E3 ubiquitin ligases such as Rnf43 and Znrf3 were found to interact with Frizzled and the Fzd/Lrp receptor complex to promote ubiquitination [65, 66]. Overexpression of these ligases causes downregulation of Frizzled receptors and Lrp5/6 at the cell surface by targeting the receptors for internalization and lysosomal degradation and causes reduction of Wnt signaling activity. Therefore, Rnf43/Znrf3 negatively regulates Wnt signaling by targeting surface Wnt receptors for degradation, which requires the Rab5-positive endocytic pathway.

Conversely, addition of R-spondin, a Wnt signal enhancer, has been shown to potentiate the Wnt signaling response [66]. R-spondin interaction with Znrf3/Rnf43 brings about membrane clearance of Znrf3/Rnf43 through endocytosis [66, 67]. Overexpression of R-spondin increases membrane levels of Fzd and Lrp by binding to the extracellular domain of Znrf3, causing inhibition of ubiquitination and the subsequent endocytosis and lysosomal degradation of the Wnt receptors. Therefore, R-spondin acts to stabilize levels of Wnt receptors at the cell surface by antagonizing E3 ubiquitin ligases. Endocytosis plays a key role in both clearance of Wnt receptors and E3 ligases from the membrane in order to control signaling.

Lgr5 was also found to have a regulatory role in Hek293 cells, providing a negative feedback loop to control Wnt/β-catenin signaling [68]. Co-stimulation of R-spondin and Wnt3a was found to promote formation of Lgr5-positive signalosomes at the membrane. Co-immunoprecipitation and FRET analysis confirmed that Lgr5 binds to Lrp6 in Lgr5-positive signalosomes. R-spondin stimulation alone did not have any significant effect on the distribution of Lrp6 within the cell; however, co-stimulation with Wnt3a increased the rate of Clathrin- and Dynamin-dependent endocytosis and promoted co-degradation of Lrp6 with Lgr5 and Fzd5 receptor. Co-stimulation caused a resultant decrease in signaling response. This suggests that over-activation of R-spondin and Wnt ligand can trigger a negative feedback loop, whereby, instead of Lgr5 promoting interaction of Wnt-receptor complexes, endocytosis of the Wnt receptors is activated. This mechanism again links endocytosis to the regulation of signaling response.

Abnormal activation of Wnt signaling, including aberrant activation of Lgr5 in the intestinal epithelium, can lead to tumorigenesis [69] and therefore, regulation of Wnt signaling via endocytosis may play a future role in cancer therapy. Tracing of cell linage showed that clones containing mutant Lgr5 isoforms, which were unable to undergo internalization, had reduced cell abundance, and thereby cell fitness [70]. This study suggests that inhibition of Clathrin-dependent endocytosis of Lgr5 has implications for controlling aberrant cell behaviors seen in some intestinal cancers. Pharmacological Lgr5 internalization inhibitors such as vacuolar-type H-ATPases were identified in a natural product library screen for further investigation.

(iv) Signal activation by stabilization of Dvl

The final proposed mechanism through which endocytosis may facilitate Wnt/β-Catenin signal activation is to stabilize Wnt-related protein Dvl2. Dvl2 is a scaffold protein that binds several Wnt signaling effectors [71]. Experiments show that an endocytic block leads to rapid degradation of the Wnt effector Dvl2 [18]. Wnt/Wg binds to and activates the seven-transmembrane receptor Fzd, which results in recruitment of the cytosolic Dvl scaffold to the membrane. Dvl2 can bind to adaptor protein β-Arrestin2 together with phosphorylated Fzd4 [72]. Dvl2 and β-Arrestin2 regulate Fzd activity by internalization and degradation. Furthermore, Dvl interacts with Rac1 to regulate the actin cytoskeleton [73]. Dvl also exists as a soluble protein that polymerizes upon activation to form discrete puncta in the cytoplasm [74]. As the Dvl2 scaffold is essential for Wnt signal transduction, its reduction could be the primary reason why a block in endocytosis reduces signaling. However, it is currently unclear how endocytosis and Dvl2 stability are linked.

The link between endocytosis and the intracellular effector Dvl2 is strengthened with evidence suggesting that there is a direct interaction between the µ2 subunit of the Clathrin-mediated endocytic complex AP2 and Dvl2 (Fig. 2(2)) [75, 76]. Dvl2 binds to one end of the elongated C-terminal domain of AP2µ2 and is recruited to Clathrin-coated pits. Interference of Dvl2–AP2µ2 binding leads to the degradation of the Dvl2 scaffold and blocks the formation and endocytosis of the signalosome [19, 48]. Why do endocytosis and AP2µ2 function promote Dvl2 stability? Recent data suggest that Dvl2 is labile and requires stabilizing binding partners [71, 77, 78]. At the plasma membrane, Dvl2 binds to, and is stabilized by, AP2µ2 [19]. Thus, AP2µ2 directly links Clathrin-mediated endocytosis to the stability of the Dvl2 scaffold.

After removal of the Clathrin coat during endocytosis, other mechanisms must exist to ensure Dvl2 stability. The cumulative evidence suggests that Dvl2 exists as a member of the multimeric transducer complex, which is not attached to intracellular vesicles [74, 79]. Indeed, recent data show the co-localization of Dvl2 with several members of the transducer complex, such as Axin1, Gsk3, and β-Catenin in the cytoplasm [3, 19, 80]. A structural analysis of the Dvl domains during Wnt activation suggests that Dvl monomers polymerize to allow signaling to β-Catenin [20]. Wnt-induced binding from Fzd to Dvl leads to translocation of the ligand–receptor complex into Clathrin-coated pits. The following Dvl-polymerization provides a platform for signalosome formation. The stability of the Dvl–Axin complex represents a possibility to protect the Dvl2 scaffold from destruction after internalization. Both proteasomal and lysosomal degradations regulate the amount of available Dvl, and the Dvl–Axin polymer formation prevents constitutive proteolytic destruction [81]. A recent report suggested that de-ubiquitination of Dvl2 leads to stabilization after activation of the Wnt pathway in HEK293T cells [82]. Thus, we hypothesize that Clathrin/AP2µ2-mediated assembly of the Dvl2 scaffold at signalosomes located in the plasma membrane and Axin1-containing transducer complexes in the cytosol both function to stabilize Dvl2 and protect it from degradation.

Following endocytosis, the Clathrin lattice is disassembled within seconds [83]. In parallel, the AP2 subunit µ2 dissociates from Dvl to be either degraded or recycled back to the membrane. Lack of AP2µ2 might lead to conformational changes of Dvl2 [20] and Axin1 [84] leading to inactivation of the Wnt/β-Catenin destruction complex (Fig. 2 (3)) [19]. The continuous block of the destruction complex promotes β-Catenin accumulation and thus Wnt/β-Catenin signaling.

## PCP-mediated endocytosis regulates cell adhesion and migration

**Fig. 3:**
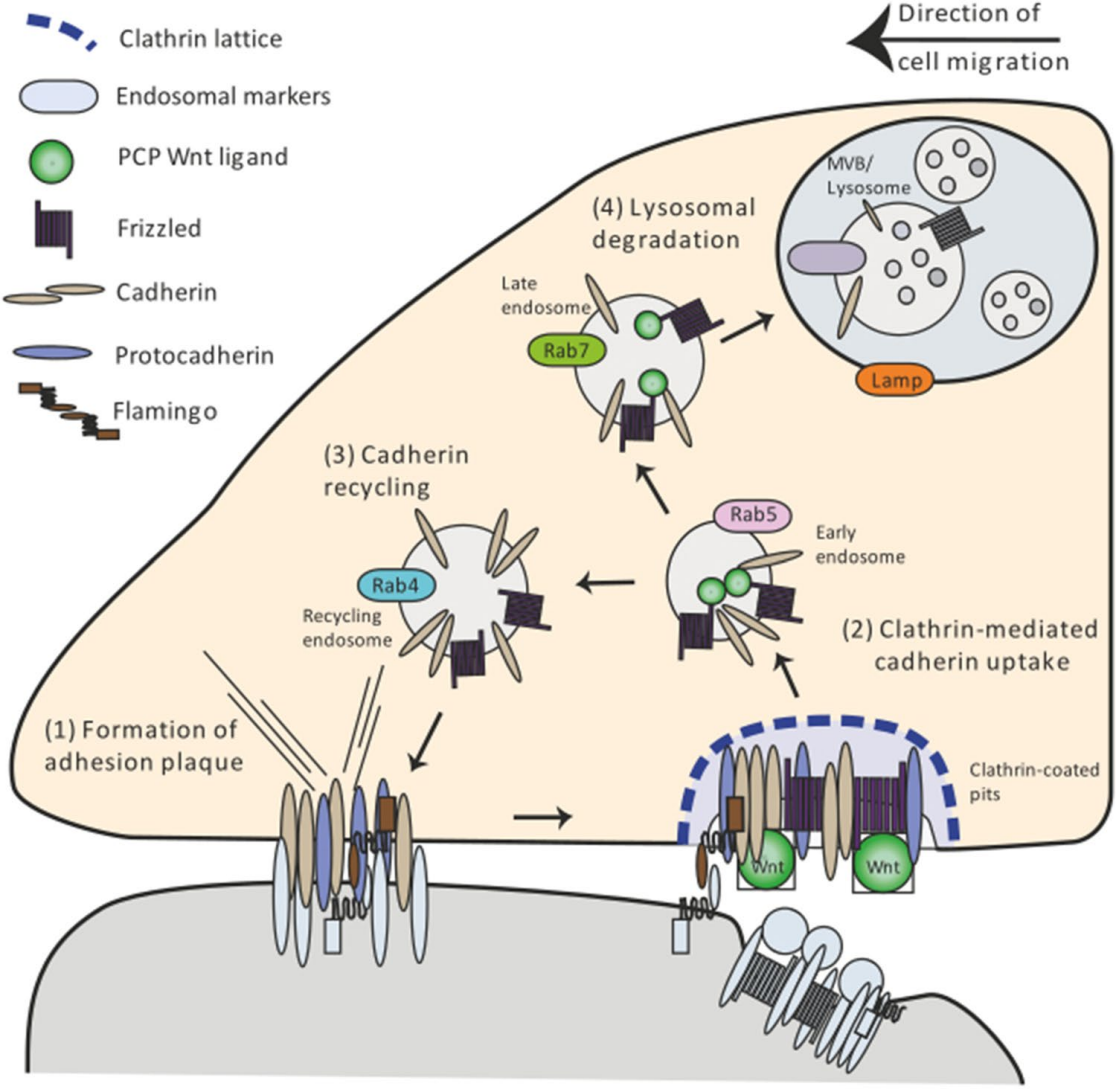
PCP signaling-regulated cell migration by endocytosis of adhesion plaques. Adhesion plaques serve as anchors to neighboring cells or substrates (1). Noncanonical Wnt signaling activates the internalization of the plaque components cadherin and protocadherin, predominantly in the rear of the cell (2). Cadherins are routed through the endocytic pathway from Rab5 early endosomes to Rab4/11 recycling endosomes (3). This allows for the assembly of new anchor points at the front of the cell. Cadherins are also routed for degradation to Rab7-positive endosomes and Lamp1 lysosomes (4). The differential rate between recycling and degradation determines the amount of Cadherin available for adhesion

The collective migration of cells during tissue morphogenesis requires dynamic modulation of cohesion between cells; this is facilitated by focal adhesion points—so-called adherence plaques [85] (Fig. 3(1)). Increasing evidence shows that the control of making and breaking these cell adherence plaques is mediated by endocytosis of adhesion molecules of the Cadherin family (Fig. 3(2)). Wnt/Planar Cell Polarity (PCP) regulates E-cadherin internalization and subsequently recycling to the cell surface (Fig. 3(3), (4)). PCP signaling has been linked to coordination of cell migration and the best studied examples are zebrafish gastrulation and development of the embryonic tracheal system in *Drosophila* [86, 87].

In zebrafish, Wnt11 regulates cell contact cohesion through promoting accumulation of the receptor Fzd7 and the cell-adhesion molecule E-cadherin at the plasma membrane together with atypical cadherin Flamingo [88]. Alteration of the function of Wnt11 or E-cadherin affects cohesive behavior of gastrulating cells [89]. E-cadherin-containing endosomes were found to co-localize with endocytic GTPases of the Rab family suggesting that E-cadherin is internalized in Rab5 early endosomes and can be recycled to the cell surface by Rab4- or Rab11-dependent endosomes [90] (Fig. 3(2), (3)). Inhibition of endocytosis caused abnormal tissue movement; which resembles the gastrulation phenotype of Wnt11 mutants. Consistently, activation of Wnt11 signaling in the zebrafish embryo increased the number of E-cadherin-containing endocytic vesicles. Recent evidence suggests that PCP signaling does not mediate internalization of Cadherins directly but rather by acting on protocadherins. Noncanonical Wnt signaling via Fzd7 together with Ror2 leads to the increased transcription of paraxial protocadherin (PAPC) in *Xenopus* [91]. Consistently, data from *Xenopus* suggest that noncanonical Wnt11 enhances endocytosis of C-cadherin by regulating PAPC to decrease C-cadherin-mediated adhesion [92]. This suggests that PCP signaling controls internalization of cadherins via protocadherins, to regulate adhesion during vertebrate gastrulation.

The process of *Drosophila* tracheal tube morphogenesis relies on cell intercalation. Abnormalities in tracheal branch cell intercalation have been detected in PCP pathway member mutants including Fzd and Dvl [93]. These PCP pathway mutants also exhibit higher levels of endogenous E-cadherin. Measurements of E-cadherin-GFP FRAP fluorescence recovery showed that PCP pathway mutants have an increased amount off stable E-cadherin at junctions, suggesting that Fzd/E-cadherin turnover from junctions is reduced. These examples demonstrate that PCP pathway members play an integral role in cadherin turnover at the membrane. Endocytosis of Wnt/β-Catenin-independent signaling together with adhesion molecules are therefore involved in a wide range of embryonic morphogenetic processes.

## Conclusion

In this paper, we have discussed three important hub points for endocytosis and the Wnt signaling network. Endocytosis plays a pivotal role in the secretion of the Wnt ligand from the source cell. In addition, increasing amounts of evidence support the hypothesis that endocytosis is required for signaling in the Wnt/β-Catenin-dependent signaling branch. Concurrently, the Wnt β-Catenin-independent pathway regulates endocytosis of cell adherence plaques, which is crucial for cell migration. This review shows that the process of Wnt signaling is inextricably intertwined with endocytosis.

To analyze specific aspects of the interdependence between endocytosis and Wnt signaling, a refinement of experimental strategies is required. This may include the distinctive manipulation of source cells and recipient cells. Furthermore, analysis of specific endocytic interactions, such as AP2µ2 with Dvl2, are required to derive a better insight into the link between single steps during the process of endocytosis and the effect on Wnt signaling. Therefore, it would be useful to resolve signaling network interactions in organelle-specific locations, such as within the endosome. Tools such as Apex (ascorbic acid peroxidase)-tagged proteins of interest have been used to capture protein interactions at specific time points following receptor activation and have been paired with quantitative proteomics to identify interactions taking place on endosomes using endosomal marker references [94]. Biosensors which allow for specific detection of signaling activation from within endosomes have been used to probe activation of internalized β2-adrenoreceptors and could also be applied to further understand Wnt signaling activation within endosomes [95].

In addition to a refined experimental strategy, the mechanisms described above highlight the relevance of cellular context in order to understand the integration of endocytosis and Wnt signaling. Wnt signaling depends on endocytosis, and thus the process of endocytosis is a major regulator of Wnt signaling. The mechanism of endocytosis changes the way Wnt signaling is secreted and integrated. This regulatory role of endocytosis on Wnt signaling may have been underestimated and should be elucidated in the future.

In general, considering the multifunctionality of endocytosis and the pivotal involvement of Wnt signaling during development and tissue homeostasis, the future may hold unexpected additional roles for endocytosis in Wnt-related processes. Thus, further research is needed to determine the functional impact of endocytosis on Wnt secretion, distribution, and function in healthy and diseased tissues.
